# Effectiveness of the QuitSure Smartphone App for Smoking Cessation: Findings of a Prospective Single Arm Trial

**DOI:** 10.2196/51658

**Published:** 2023-12-29

**Authors:** Apurvakumar Pandya, Mythri K S, Shweta Mishra, Kriti Bajaj

**Affiliations:** 1 Parul Institute of Public Health Parul University Vadodara India; 2 Indian Institute of Public Health Gandhinagar India; 3 RapidKart Online Pvt Ltd Mumbai India

**Keywords:** smoking, nicotine dependence, smoking cessation, QuitSure app, smartphone application, mHealth, mobile health, app, apps, application, applications, nicotine, smoke, smoker, quit, quitting, cessation, abstinence, mobile phone

## Abstract

**Background:**

Digital therapies, especially smartphone apps for active and continuous smoking cessation support, are strongly emerging as an alternative smoking cessation therapy. In the Indian context, there is a growing interest in the use of app-based smoking cessation programs; however, there is limited evidence regarding their effectiveness in achieving long-term continuous abstinence.

**Objective:**

This study aimed to evaluate the long-term abstinence effect (up to 30-d abstinence postprogram completion) of a smartphone app, QuitSure, for smoking cessation in active smokers from India.

**Methods:**

In this prospective single-arm study, participants who signed up for the QuitSure app were enrolled in this study. The primary end point was the prolonged abstinence (PA) rate from weeks 1 to 4 (day 7 to day 30). Furthermore, data for withdrawal symptoms, relapse reasons, and reasons for not continuing the program were also assessed.

**Results:**

The quit rate was calculated considering only the participants who followed up and completed the survey sent to them (per protocol) at day 7 and at day 30, respectively. The PA rate at day 7 was found to be 64.5% (111/172; 95% CI 56% to 72%), and the PA rate at day 30 was found to be 55.8% (72/129; 95% CI 45% to 65%). Within the 7-day abstinence period, 60.4% (67/111) of the participants did not have any withdrawal symptoms. The most common mild withdrawal symptoms were mild sleep disturbance (21/111, 18.9%), mild digestive changes (19/111, 17.1%), and coughing (17/111, 15.3%). Severe withdrawal symptoms were rare, with only 5.4% (6/111) experiencing them. For those achieving 30-day postprogram abstinence, 85% (61/72) had no mild withdrawal symptoms, and 99% (71/72) had no severe withdrawal symptoms. Among successful quitters at day 7, a total of 72.1% (80/111) reported minimal to no cravings, which increased to 88% (63/72) at day 30. Furthermore, 78% (56/72) of those with PA at day 30 reported no change in weight or reduced weight. Among participants experiencing relapse, 48% (28/58) cited intense cravings, 28% (16/58) mentioned facing a tragedy, and 26% (15/58) reported relapsing due to alcohol consumption. The PA rates as a result of the QuitSure program were found to be better than those reported in the results of other smoking-cessation app programs’ studies.

**Conclusions:**

The QuitSure app yields high PA rates and ameliorates symptoms associated with smoking cessation. In order to obtain conclusive evidence regarding the effectiveness and efficacy of the QuitSure program, future research should include appropriate control measures. Nevertheless, the QuitSure program can serve as a valuable adjunct to a conventional smoking cessation treatment program to aid sustained abstinence.

## Introduction

### Background

Tobacco smoking remains one of the leading causes of preventable death worldwide [1]. According to the Global Adult Tobacco Survey India, 2016 to 2017, nearly 267 million adults in India use tobacco in one way or the other, of which 120 million are smokers, making India home to 12% of the world’s smokers [2]. Of the total Indian adults who smoke, only a minority take advantage of evidence-based approaches to smoking cessation.

The most effective tobacco cessation programs require personalized human intervention combined with costly pharmaceutical supplementation, making them unaffordable or inaccessible to most tobacco users [3,4]. Thus, digital therapies, delivered through smartphone apps, offer a promising alternative to these traditional methods [5-8].

The reach of smoking cessation apps is rising rapidly, greatly owing to the growing ownership of smartphones. As of 2023, a total of 6.92 billion people, representing around 86.11% of the world’s population, have smartphones [9]. Therefore, there is a tremendous opportunity to explore the effectiveness of smartphone apps to reverse nicotine dependence.

### Smoking Cessation and Smartphone Apps

We reviewed existing smartphone apps for smoking cessation. Very few mobile apps have clinical evidence. Apps that have undergone clinical trials [10-17] to evaluate their effectiveness in aiding smoking cessation interventions exhibit various limitations. Recent evidence from observational [18,19] cohort studies [20,21] also highlights promising results of a smartphone-based smoking cessation program. The majority of these interventions are prohibitively expensive (US $299 [₹23,612] or more; at an exchange rate of ₹78.97 per US dollar as of June 30, 2022), inaccessible to individuals, not available in English, face low acceptance in the market, or lack updates for over a year [22,23].

The evidence of long-term continuous abstinence using app-based smoking cessation programs is limited. A systematic review [22] published in 2016 analyzed 28 randomized controlled trials that evaluated the effectiveness of mobile smoking cessation apps. The review found that mobile apps can increase the chances of quitting smoking compared to no intervention or minimal support. However, the quality of evidence was considered low to moderate, indicating the need for further research. Another systematic review conducted on the same research topic by Chu et al [23] in 2021 also concluded similar findings. The study reported a mean abstinence rate of 33.9% of 7 single-arm trials. Additionally, another review of smoking cessation apps, which evaluated 98 available apps, found that 33.7% (33/98) of them had less than 10,000 downloads, indicating low market acceptance [24]. Furthermore, a study by Seo et al [25] discovered that out of 104 smoking cessation apps in their investigation, a substantial 88.5% (n=92) had not received updates in over a year. Lastly, a noteworthy 48.1% (n=50) of these apps were paid, limiting user access for those who preferred to try a demo before committing to the program [25]. We did not find any Indian study on smartphone-based smoking cessation apps based in India.

This study attempts to understand the prolonged abstinence (PA) rates at day 7 and day 30 of an Indian smoking cessation app—QuitSure. In this study, we aim to present the report of abstinence at 2 time points: 7-day and 30-day postprogram completion.

### Description of the QuitSure App

**Figure 1:**
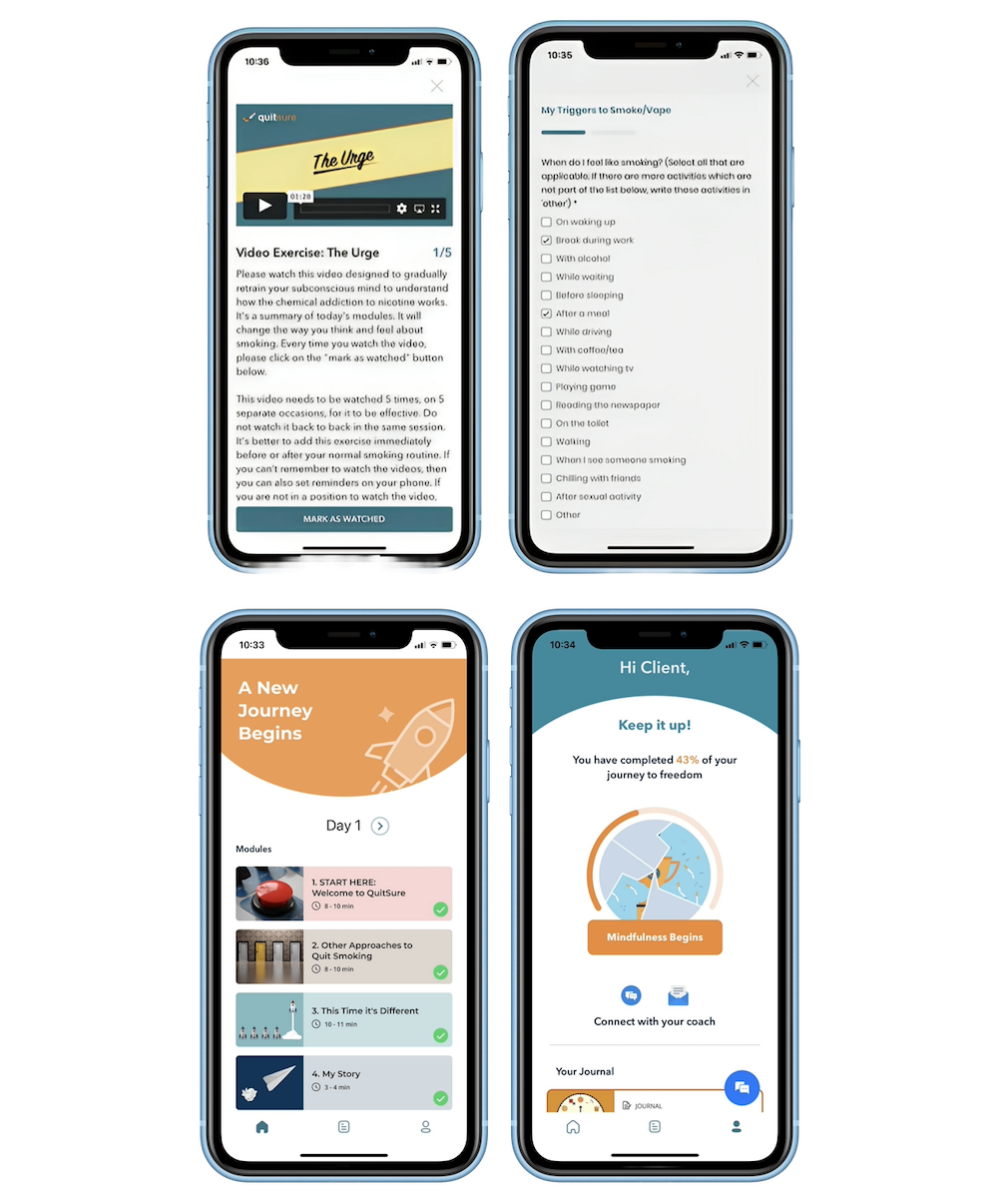
Features of QuitSure app.

The QuitSure app (Figure 1) is a personalized 6-day, do-it-yourself program designed to quit smoking easily without any cravings and weight gain after quitting. It has 6-10 hours of focused reading and videos that are required to complete the program. When users open the app, they are assessed on their smoking habits, including factors such as cigarette expenditure and previous quit attempts. They are then directed to the main menu of the app. Key features of the app include a quit plan, mindfulness activities, motivational content, and coaching. The program requires no lifestyle changes.

The primary objective of the QuitSure app is to offer an accessible solution for daily smokers looking to quit. Unlike pharmacotherapy, which is often criticized for being expensive and having side effects such as nausea and discomfort, the QuitSure app does not recommend pharmacotherapy. Instead, it focuses on helping smokers quit through methods rooted in cognitive behavioral therapy (CBT), mindfulness, positive psychology, and in-app chat-based support. The QuitSure app is regularly updated, approximately once every 3 weeks since its launch.

CBT is integrated into the program to assist users in challenging their misconceptions and beliefs about their cravings and urges to smoke. It helps users restructure these beliefs by guiding them to adopt a more rational and suitable way of thinking and feeling about their addiction. The app incorporates mindfulness and offers in-text and video activities that encourage users to practice breathing techniques while smoking. When they smoke, they are asked to be mindful of the taste, the smoke, and their thoughts.

At the end of the program, users are expected to upload a picture of their final cigarette and write a script explaining why they have decided to give up smoking as part of a “quitting ceremony” activity. This is their designated official “quit date.”

## Methods

### Design

A pre-post single-arm, digital trial design was used. This study aimed to assess the effectiveness of a digital therapeutic app designed for facilitating smoking cessation among adult participants who own an Android or iOS smartphone.

The study population was Indian adult participants who are everyday smokers and have signed up for the QuitSure program.

The inclusion criteria were as follows:

- Adult participants (aged 18 years or older) who have signed up for the QuitSure program
- An everyday smoker of cigarettes: should *currently* smoke at least 1 cigarette per day with an average rate of 1-5 cigarettes per day (indicating sufficient dependence on nicotine) and have smoked at least 100 cigarettes (indicating sufficient exposure of the body to nicotine) in the past 3 and half months
- Having at minimum proficiency in written and spoken English
- Have daily access to a smartphone and the internet

The exclusion criteria were as follows:

- Have already quit smoking and are currently *not* smoking
- If a doctor has advised the participant not to quit “cold turkey”
- Participating in any other smoking cessation intervention (eg, nicotine patches and medications)
- Anyone not willing to participate
- Anyone with severe medical conditions

### Recruitment

From December 2022 to February 2023, potential participants were recruited through advertisements on Facebook and Google and directly via the QuitSure app. They were given a link to register for this study. Smokers who signed up for the QuitSure program were given the option to participate in this study by filling out the attached “Participant Information Sheet” and “Informed Consent Form.” Those who consented to participate in this study and fell within the eligibility criteria described above were considered as the final intervention participant group.

Participants who screened as eligible, completed consent, and filled out baseline measures (n=849) were provided a program fee waive-off voucher and invited to participate in this study. Of these, 493 of them activated their vouchers and registered for the QuitSure program. They were provided the QuitSure program for free on the app with the help of these vouchers. To maximize retention, participants were compensated with an entry into a raffle with the chance to win a US $50 (₹3949) Amazon gift voucher for completing this study and all surveys.

Based on a review of previously published studies on retention rate, we anticipated that approximately 40% would not complete the program and an additional 20% to be lost to follow-up postprogram completion. To achieve a final data set of 100 participants, we recruited an initial data set of 493 eligible smokers who enrolled in this study and registered for the QuitSure program. Of these, 210 completed the program, and 129 completed the follow-up web surveys 30 days after the program and were considered the final per-protocol data set.

### Web-Based Survey

All questionnaires were prepared on the HIPAA (Health Insurance Portability and Accountability Act)–compliant Jotform platform (Jotform Inc) [26], and data are available in a dedicated, access-controlled Google account (GA). Only primary researchers had access to original raw data, which were deidentified and transferred to Microsoft Excel sheets for the purpose of analysis. This study had 2 phases: the preintervention and postintervention phases. In the preintervention phase, participants provided demographic data, smoking history, and motivation to quit.

In the postintervention phase, data were collected after the quitting ceremony, assessing the use of cessation aids, abstinence, withdrawal symptoms, and cravings. Longitudinal data were gathered at day 7 and day 30 to track abstinence and withdrawal symptoms. Participants also shared feedback on the QuitSure program and their willingness to use it again.

### Study Variables

The primary outcome was to detect smoking cessation; the postprogram completion follow-up survey assessed 7-day and 30-day abstinence.

The Society for Research on Nicotine and Tobacco Subcommittee on Biochemical Verification recommends that biochemical confirmation is unnecessary in population-based studies with limited face-to-face contact and studies where the optimal data collection methods are through the mail or telephone [27]. Therefore, self-reported smoking is a standard method for assessing the efficacy of low-intensity interventions [28-32] and was used in this study.

The secondary outcomes were the motivation level, craving levels, withdrawal symptoms, reasons for relapse, and qualitative feedback on the intervention, which were measured through the postprogram completion follow-up survey. An analysis was also conducted for the reasons for the discontinuation of the program by program incompleters.

### Ethical Considerations

This study received clearance from the Parul University’s Institutional Ethics Committee for Human Research (approval PUIECHR/PIMSR/00/081734/4714; dated July 15, 2022). Smokers who signed up for the QuitSure program were given the option to participate in this study by providing digitally signed consent. All study-related data were stored in a dedicated, access-controlled GA, and all changes made to the data were logged and auditable. Questionnaires were prepared on the HIPAA-compliant Jotform platform, and participant responses were directly entered into noneditable Google Sheets in the GA.

This study maintained the confidentiality of participants’ information and data. Only the primary researcher had access to the original raw data, which was deidentified in full compliance with the strictest HIPAA norms. Poststudy completion, all data were securely transferred to a dedicated, access-controlled Amazon Web Services S3 bucket for a period of 5 years. Participants received full access to the QuitSure program for free. They were compensated with an entry into a raffle with the chance to win a US $50 (₹3949) Amazon gift voucher for completing this study and all surveys.

### Statistical Analysis

For the primary aim, descriptive statistics were used to estimate smoking cessation and the impact of program completion. Consistent with complete case analytic methods, only those who responded to questions about their smoking status in the outcome survey were included in the final analyses and discussion (per protocol). We deliberately avoided making assumptions that nonrespondents were still smoking (ie, treating missing data as smoking cases). This is because previous research has shown that such imputation can introduce biases in the estimates of effect size. Depending on the actual smoking cessation rate among participants, using this worst-case scenario assumption can either result in reduced statistical power and increased likelihood of type II errors or lead to an underestimation of variability and increased likelihood of type I errors [32,33]. However, for the sake of completeness and comparison, we have included missing-equals-smoking numbers in the data tables.

## Results

### Participant Characteristics

Participants who completed the consent form completed digital baseline survey that assessed demographic and smoking characteristics. Table 1 shows baseline characteristics of study participants. We administered a follow-up survey at 7-day and 30-day postprogram completion.

Of the 493 study participants who registered for the QuitSure program, 57.4% (283/493) left the program midway, while 42.6% (210/493) completed the program. Of these program completers, 61.4% (129/210) completed the required web survey at 30-day postprogram completion.

**Table 1:** Baseline characteristics of the enrolled participants (N=493).

Characteristics		Participants, n (%)^a^
**Gender**		
	Men	467 (94.7)
	Women	25 (5.1)
	Other	1 (0.2)
**Age group (y)**		
	18-24	175 (35.5)
	25-34	259 (52.5)
	35-44	49 (9.9)
	45-59	10 (2)
**Level of education**		
	High school or technical course	52 (5.1)
	Bachelor’s degree	300 (60.8)
	Master’s degree	129 (26.2)
	Doctorate degree	11 (2.2)
	None of the above	1 (0.2)
**Occupation**		
	Student	130 (26.3)
	Salaried	255 (51.7)
	Business or freelance	77 (15.6)
	Unemployed	31 (6.3)
**Average number of cigarettes per day**		
	1-5	148 (30)
	6-10	201 (40.8)
	11-15	79 (16)
	>16	69 (13.2)
**Level of motivation**		
	Highly motivated	263 (53.4)
	Moderately motivated	187 (37.9)
	Low motivation	38 (7.1)
	No motivation	05 (1)
**English-language ability**		
	Conversational	88 (17.8)
	Fluent (native)	114 (23.1)
	Fluent (nonnative)	199 (40.4)
	Proficient	92 (18.7)

^a^All participants had Indian nationality.

### Effectiveness of the QuitSure App on Smoking Cessation

For the purpose of this study, we primarily considered the participants who adhered to this study’s protocol as our final data set for study analysis. Table 2 presents that at 30-day postprogram completion, 85.3% (110/129) reported complete cessation or a substantial reduction in smoking levels as compared to what they smoked prior to starting the program. Further, 55.8% (72/129) had achieved 30-day PA (0 cigarettes smoked in the 30 days since program completion).

This study assessed the PA rates among participants at 7-day and 30-day postprogram completion. On day 7, a total of 81.9% (172/210) of program completers reported their smoking status, with 64.5% (111/172) achieving 7-day PA (per-protocol analysis). At day 30, a total of 61.4% (129/210) of participants reported their smoking status, with 55.8% (72/129) reporting PA for the entire 30 days.

Of the 129 respondents at 30-day postprogram completion, 57 (44.2%) reported relapses. Of these, 35% (20/57) had reported extreme reduction of ≤2 cigarettes total over the 30-day period, while 32% (18/57) reported a substantial reduction of 30% to 95% compared to before starting the program.

For missing-equals-smoking analysis, nonresponders of the web-survey were assumed to have relapsed or failed to quit after doing the program. All the 210 participants who completed the program were considered to assess quit smoking rates. The missing-equals-smoking PA rate for 7-day and 30-day follow-up were 52.9% (111/210) and 34.3% (72/210), respectively.

We performed chi-square tests to examine the impact of education level, gender, age, English-language ability, income, or motivation levels on quit rate via the QuitSure program. The analysis revealed no statistically significant association of any of these demographic variables—educational level (*P*=.23); gender (*P*=.18); age (*P*=.10); English-language ability (*P*=.42); income levels (*P*=.90); and motivation level (*P*=.74)—on cessation outcomes.

**Table 2:** Program cessation and reduction outcomes.

Outcome and time point		Participants, n/N (%)		Value (%), 95% CI
**Prolonged abstinence**				
	Day 7 (PP^a^)	111/172 (64.5)	56-72	
	Day 7 (MES^b^)	111/210 (52.9)	44-61	
	Day 30 (PP)	72/129 (55.8)	45-65	
	Day 30 (MES)	72/210 (34.3)	27-41	
**Reduction in smoking**				
	Day 7 (PP)	44/172 (25.8)	19-32	
	Day 7 (MES)	44/210 (20.5)	13-23	
	Day 30 (PP)	38/129 (29.5)	21-38	
	Day 30 (MES)	38/210 (18.1)	15-27	

^a^PP: per protocol analysis, where dropouts were not considered for the analysis.

^b^MES: missing equals smoking, where dropouts were considered to be program failures.

### Withdrawal Symptoms After Quitting

**Figure 2:**
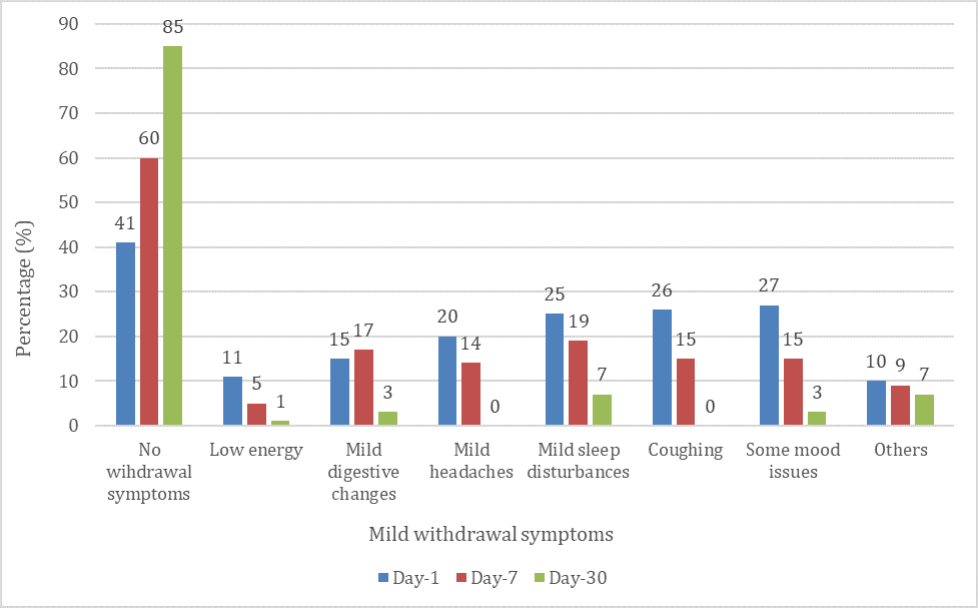
Mild withdrawal symptoms reported at 1-day, 7-day, and 30-day postprogram completion by those who achieved prolonged abstinence.

**Figure 3:**
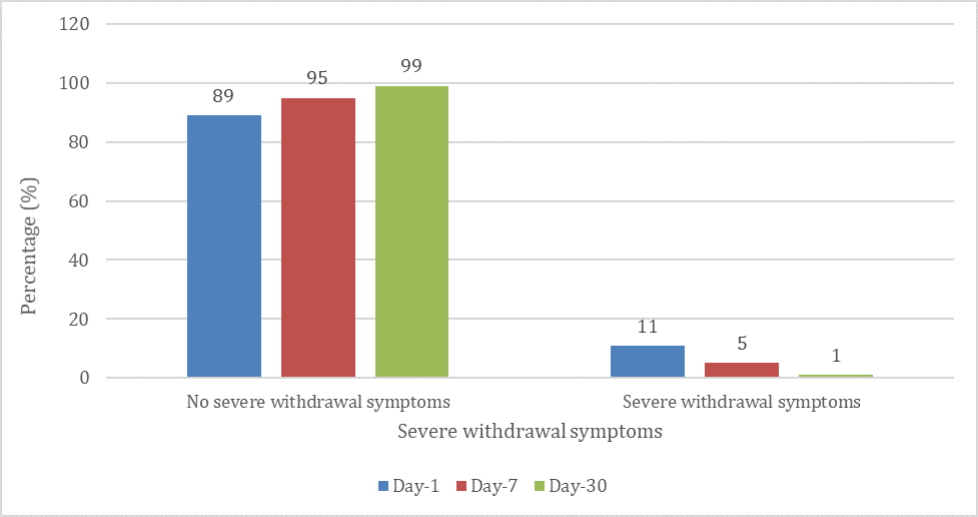
Severe withdrawal symptoms reported at 1-day, 7-day, and 30-day postprogram completion by those who achieved prolonged abstinence.

Among the participants who had achieved 7-day PA, 60.3% (67/111) did not experience any withdrawal symptoms at all in the 7 days after completing the program. Figure 2 illustrates that the most common mild withdrawal symptoms observed were mild sleep disturbance 18.9% (21/111), mild digestive changes 17.1% (19/111), and coughing 15.3% (17/111). Figure 3 shows severe withdrawal symptoms such as strong chest pain, dizziness, nausea, and insomnia among successful quitters. Only 5.4% (6/111) of them experienced any such severe withdrawal symptoms. All these reactions were further reduced among participants who achieved 30-day PA after the program, with 85% (61/72) experiencing no mild withdrawal symptoms and 99% (71/72) experiencing no severe withdrawal symptoms.

### Cravings and Weight Changes After Quitting

Table 3 presents cravings of participants who achieved PA at 1-day, 7-day, and 30-day postprogram completion . In all, 72.1% (80/111) of successful quitters reported experiencing minimal to no cravings at 7-day postprogram completion, while 88% (63/72) reported having minimal to no cravings at 30-day postprogram completion.

**Table 3:** Cravings reported by the participants who achieved PA^a^ at 1-day, 7-day, and 30-day postprogram completion.

Cravings	1-Day PA (n=133), n (%)	7-Day PA (n=111), n (%)	30-Day PA (n=72), n (%)
No craving or minimal craving	57 (42.8)	80 (72.1)	63 (87.5)
Mild craving	53 (39.8)	24 (21.6)	9 (12.5)
Moderate craving	17 (12.8)	6 (5.4)	0 (0)
High craving	6 (4.5)	1 (0.9)	0 (0)

^a^PA: prolonged abstinence.

Table 4 presents weight changes of participants who achieved PA at 7-day and 30-day postprogram completion . At 30-day postprogram completion, 78% (56/72) of those who had achieved PA reported no change in weight or reduced weight since completing the program.

**Table 4:** Weight changes reported by participants who achieved PA^a^ at 7-day and 30-day postprogram completion.

Weight changes	7-Day PA (n=111), n (%)	30-Day PA (n=72), n (%)
Reduced	10 (9)	10 (13.9)
Remained the same	82 (73.9)	46 (63.9)
Increased	19 (17.1)	16 (22.2)

^a^PA: prolonged abstinence.

### Factors Contributing to Relapse

Among the participants who experienced relapse and were considered as program failures, 58 individuals provided feedback on the reasons that led to their relapse. Table 5 shows causes of relapse. Out of 58 participants, 48% (n=28) reported intense cravings for cigarettes, 28% (n=16) reported facing a tragedy, and 26% (n=15) reported the consumption of alcohol as reasons of relapse.

**Table 5:** Reasons for relapse stated by study participants.

Reasons for relapse	Participant (N=58), n (%)
Bad cravings	28 (48)
Faced a tragedy or personal reasons	16 (28)
Gave in while drinking alcohol	15 (26)
Overconfidence	13 (22)
Self-destruction	10 (17)
Physical withdrawal symptoms	6 (10)
Did not do the program properly	5 (9)
Smoking deceptions	3 (5)
Stress	3 (5)
Peer pressure	3 (5)

### Reasons for the Discontinuation of the Program

Of the 283 participants who left the program midway, 66 (23.3%) reported their reasons for doing so. Further, 2 participants reported that they had already quit smoking with the help of the app and were thus excluded from the analysis, making the final number 64. In all, 48% (31/64) of the participants accorded the reason for the discontinuation of the program to their busy schedule. Further, 31% (20/64) of participants expressed fear of success. Table 6 provides details of the reasons participants attributed to their discontinuation of the program.

**Table 6:** Reasons for discontinuing the program.

Reasons for discontinuing	Participants (n=64), n (%)
Busy, not able to spare time	31 (48)
Fear of success	20 (31)
Family, personal, or logistical issues	13 (20)
Technical issues in app	2 (3)
Not enjoying the program	1 (2)
App helped me cut down and I didn’t care to quit	2 (3)

## Discussion

### Principal Findings

This study determined the quit rate and abstinence of a smartphone app for smoking cessation (QuitSure). This study advances the evidence base for smartphone apps for smoking cessation. Prior randomized clinical trials in a 2019 Cochrane review ranged in sample size from 49 to 1599 and had a weighted mean retention rate of 55%. This trial had a slightly higher retention rate (ie, 58% vs 55%). The retention rate of 58% was higher than the 54% typically obtained in web-based cessation trials [30,31] but quite less than the retention rates (85% and 84%) reported in Bricker et al [34] and Hutton et al [35], respectively.

The self-reported 30-day abstinence rates of individuals using smartphone apps included in the Cochrane review ranged from 4% to 18%, whereas this study reported a 30-day abstinence rate of 55.8%. Bricker et al [34] reported a 30-day abstinence rate of 28% in a recent single-arm trial, indicating a higher PA rate of the QuitSure smoking cessation program. This indicates that QuitSure is more advanced than other smartphone-based smoking cessation apps.

To assess the efficacy of the QuitSure app, the 6-month, 9-month, and 12-month abstinence rates were examined. Prospective research should examine theoretical processes as well as specific features listed in the *Introduction* section to unveil why QuitSure was the more efficacious intervention.

According to the findings from this study, more than half (283/493; 57.4%) the participants did not complete the program. This was found to be higher than what was reported in Bricker et al [34]. It suggests that a key to making QuitSure more effective for smoking cessation could be to increase program engagement and program completion—as quit rates were much higher among completers.

The secondary outcomes explored the withdrawal symptoms, cravings experienced by participants after achieving abstinence, reasons for relapse, and qualitative feedback on the intervention. We also explored the reasons why participants discontinued the program. A notable finding of this study was that 60.3% (67/111) of participants reported no withdrawal symptoms at 7 days of abstinence, increasing to 85% (61/72) at 30 days. This contrasts with previous research indicating that 46.3% of smokers experience substantial withdrawal symptoms upon quitting [36-38]. Only 5.4% (6/111) and 1% (1/72) of participants experienced severe withdrawal symptoms at 7 days and 30 days, respectively. The program’s emphasis on psychological approaches, such as mindfulness techniques and CBT, likely contributed to its effectiveness in reducing withdrawal symptoms and improving cessation rates. Withdrawal symptoms are known to be a major factor in relapse [39], highlighting the program’s impact on long-term quitting success.

Weight gain after quitting smoking has been a great concern as nicotine is known to suppress appetite and increase metabolism [40]. Previous research found that 35.4% of quitters experienced significant weight gain [41]. However, in this study, more than half (46/72, 64%) the participants maintained their weight, and only 22% (16/72) reported weight gain. QuitSure’s success in maintaining or reducing weight can be attributed to controlled thought patterns associated with food, hunger, and smoking in order to prevent excessive food intake after quitting.

When it comes to reasons for relapse, 48% (28/58) of participants reported that their relapse was triggered by intense cravings for cigarettes. Meanwhile, 28% (16/58) of participants reported that facing a tragedy triggered their relapse, while 269% (15/58) of participants reported relapsing when they consumed alcohol and were unable to resist the urge to smoke. At present, the QuitSure program currently does not address postprogram cravings management. To prevent relapse, it is suggested that QuitSure monitor craving levels after the program and provide additional content and counseling for individuals struggling with cravings. Additionally, incorporating support for individuals facing tragedies and strategies for handling smoking triggers related to alcohol consumption would further enhance the program’s effectiveness in preventing relapse.

QuitSure does not currently incorporate nicotine replacement therapy in its protocol, despite its recommendation by the World Health Organization, Centres for Disease Control and Prevention, and National Institute for Health and Care Excellence as an effective complement to counseling [3,41,42]. Nicotine replacement therapy has shown to significantly enhance success rates in psychology-based smoking cessation programs [43] by reducing withdrawal symptoms and cravings. To further improve efficacy, QuitSure could consider incorporating a phased-out nicotine replacement regimen after the program. While existing literature, such as the study by Bricker et al [34], demonstrates the efficacy of smoking cessation apps, this study offers insights into specific program features and outcomes that can be invaluable for enhancing cessation strategies.

### Limitations and Strengths of This Study

We acknowledge the limitations as well as strengths of this study. First, a single-arm prospective trial was conducted. Without a control group or placebo, it makes it difficult to determine whether the observed outcomes are due to the QuitSure program or other factors. Second, this study collected self-reported smoking, motivation, and relapse data, which may introduce socially desirable response or recall bias. Third, we could not calculate the association between demographic variables, smoking quit rate, smoking reduction, and abstinence rate. Fourth, the sample used in this study may not be representative of all smokers, limiting its generalizability. Lastly, this study did not assess what users liked about the app, which could be included in future studies to expand QuitSure program’s implications. It is also worth noting that our study shares some limitations similar to other existing studies, such as high dropout rates [17,19]. We acknowledge the challenge posed by dropout rates in smoking cessation studies and recognize the potential to magnify cessation rates as a limitation.

Notwithstanding the numerous limitations of this study, this investigation holds significant value due to its pioneering nature as the first study of its kind to examine an Indian smoking cessation smartphone app within an Indian demographic, as far as our current knowledge extends. This study provides promising evidence on high smoking quit rates among those who have completed the program and important insights on how to improve program engagement and completion rates.

### Conclusion

The QuitSure app has the potential to enhance sustained abstinence rates and ameliorate symptoms associated with smoking cessation. Enhancing program engagement and completion rates can lead to further improvements in outcomes. In order to obtain reliable and conclusive evidence regarding the effectiveness and efficacy of the QuitSure program, future research should incorporate appropriate control measures. The QuitSure program can be served as a valuable adjunct to a conventional smoking cessation treatment program to aid in sustained abstinence.

## Abbreviations

CBT: cognitive behavioral therapy
GA: Google account
HIPAA: Health Insurance Portability and Accountability Act
PA: prolonged abstinence

## Data Availability

The data sets generated and analyzed during this study are not publicly available due to ethical concerns but are available from the corresponding author on reasonable request.
